# Multicenter surveillance study of surgical site infection and its risk factors in radical resection of colon or rectal carcinoma

**DOI:** 10.1186/s12879-019-4064-6

**Published:** 2019-05-14

**Authors:** Mingmei Du, Bowei Liu, Meng Li, Jingui Cao, Ding Liu, Zhigang Wang, Qiongshu Wang, Pengyun Xiao, Xinling Zhang, Yanxin Gao, Hua Zeng, Jing Yang, Xiaoli Xu, Yi Huang, Qun Zhang, Bo Zhang, Wei Chen, Jieran Shi, Shanhong Fan, Fuxiang Zhang, Jinyan Yang, Huining Yang, Zhaoxia Ding, Haifeng Li, Sha Xiao, Suping Ran, Hongyan Zhai, Fang Wang, Yubin Xing, Jijiang Suo, Yunxi Liu

**Affiliations:** 1 Department of Infection Management and Disease Control, Chinese PLA General Hospital, 28 Fuxing Rd, Haidian District, Beijing, 100853 China; 2 Department of Hematology, Chinese PLA General Hospital, Beijing, China; 3 Department of Infection Control, Air Force General Hospital, Beijing, China; 4 Department of Infection Control, Daping Hospital, Chongqing, China; 5 Department of Infection Control, The Army General Hospital, Beijing, China; 6 Department of Infection Control, Wuhan General Hospital, Wuhan, China; 7 Department of Disease Prevent and Control, Jinan General Hospital, Jinan, China; 8 Department of Infection Control, Lanzhou General Hospital, Lanzhou, China; 9 Department of Disease Prevent and Control, Urumqi General Hospital, Urumqi, China; 10 Department of Infection Control, Fuzhou General Hospital, Fuzhou, China; 11 Department of Infection Control, ShenYang General Hospital, ShenYang, China; 12 Department of Infection Control, Nanjing General Hospital, Nanjing, China; 13 Department of Infection Management, Changhai Hospital, Shanghai, China; 14 Department of Infection Management, Changzheng Hospital, Shanghai, China; 15 Department of Infection Control, Xinan Hospital, Chongqing, China; 16 Department of Infection Control, Xinqiao Hospital, Chongqing, China; 17 Department of Infection Control, Xijing Hospital, Xian, China; 18 Department of Infection Control, Tangdu Hospital, Xian, China; 19 Department of Infection Control, General Hospital of the PLA Rocket Force, Beijing, China; 20 Department of Infection Management and Disease Control, Hainan Hospital, Sanya, China; 21 Department of Infection Control, The Armed Police General Hospital, Beijing, China; 22 Department of Infection Control, 81 Hospital of PLA, Nanjing, China; 23 Department of Infection Control, 202 Hospital of PLA, Shenyang, China; 24 Department of Infection Control, 211 Hospital of PLA, Haerbin, China; 25 Department of Infection Control, 306 Hospital of PLA, Beijing, China; 26 Department of Infection Control, 307 Hospital of PLA, Beijing, China; 27 Department of Infection Control, 309 Hospital of PLA, Beijing, China

**Keywords:** Surgical site infection, Radical resection of colon carcinoma, Radical resection of rectal carcinoma, Prospective surveillance, Multicenter study

## Abstract

**Background:**

Colorectal surgery is associated with high rates of surgical site infection (SSI). We investigated SSI in radical resection of colon or rectal carcinoma and its epidemiological distribution in 26 hospitals in China.

**Methods:**

We conducted prospective surveillance of patients who underwent radical resection of colon or rectal carcinoma in 26 selected hospitals from January 2015 to June 2016.An information system monitored all of the surgical inpatients. Infection control professionals observed the inpatients with suspected SSI who had been screened by the system at the bedside. The infection status of the incisions was followed up by telephone 1 month after the operation.

**Results:**

In total, 5729 patients were enrolled for the two operations; SSIs occurred in 206 patients, and the infection rate was 3.60%. The incidence of SSI after radical resection of rectal carcinoma (5.12%; 119/2323) was 2.1 times higher than that after radical resection of colon carcinoma (2.55%; 87/3406) (*P* < 0.0001). Additionally, in the colon versus rectal groups, the rate of superficial incisional SSI was 0.94% versus 2.28% (*P* < 0.0001), the rate of deep incisional SSI was 0.56% versus 1.11% (*P* = 0.018), and the rate of organ space SSI was 1.06% versus 1.72% (*P* = 0.031), respectively. The most common pathogens causing SSIs after radical resection of colon carcinoma were *Escherichia coli* (21/38) and *Pseudomonas aeruginosa* (5/38). *Escherichia coli* (24/65) and *Enterococcus* spp. (14/65) were the two most common pathogens in the rectal group. The multivariate logistic regression analysis showed that only the operating time and number of hospital beds were common independent risk factors for SSIs after the two types of surgery.

**Conclusion:**

This multicenter study showed that there were significant differences in the incidence of SSIs, three types of SSIs, and some risk factors between radical resection of colon carcinoma and rectal carcinoma.

## Background

The occurrence of surgical site infection (SSI) can lead to psychological trauma, prolong postoperative recovery, and increase the disease burden and mortality. Postoperative SSI is one of the most common complications of radical resection of colorectal carcinoma, and the incidence reportedly ranges from 8 to 30% in different studies [1, 2].The Japanese researcher Tsuyoshi Konishi [3] suggested that for colorectal surgery, a distinction should be made between colonic and rectal surgery because of the differences in the incidence of SSI, infection types, and specific risk factors. The occurrence of most SSIs is preventable; therefore, multicenter SSI surveillance and evaluation protocols that distinguish the type of surgery and disease are necessary in the field of colorectal surgery. Few multicenter surveillance studies of infections in colorectal surgery have been carried out in China. In the present study, we performed multicenter surveillance of radical resection of colon and rectal carcinoma in 26 hospitals [codes of procedures and diseases were restricted in detail according to the International Classification of Diseases, 9th revision; Clinical Modification of Operations and Procedures (ICD-9-CM-3) and International Classification ofDiseases,10th revision (ICD-10), respectively]. We herein report the incidence of and main risk factors for SSIs in radical resection of colon and rectal carcinoma in China to improve prevention and control of SSIs in these operations.

## Methods

### Data sources

From January 2015 to June 2016, 26 hospitals in 14 cities in China participated in the SSI surveillance project. All hospitals were tertiary general hospitals and adopted the same information system to monitor the SSIs. More than five inpatients underwent radical resection of colon or rectal carcinoma in each hospital per month. Training sessions were held to standardize the definition of SSIs, the SSI surveillance methods by the information system, the data collection, the wound secretion culture, and the follow-up method. In total, 5729 patients with two types of disease (selected codes from ICD-10) treated by two types of surgery (selected codes from ICD-9-CM-3) were monitored: 3406 underwent radical resection of colon carcinoma, and 2323 underwent radical resection of rectal carcinoma. The ICD-9-CM-3 codes for radical resection of colon carcinomawere 45.73, 45.74, 45.75, 45.76, and 45.79; the ICD-9-CM-3 codes for radical resection of rectal carcinoma were 48.5, 48.61, 48.62, 48.63, 48.64, 48.65, and 48.69.

### Surveillance method

An information system called the Real-Time Nosocomial Infection Surveillance System (RT-NISS) monitored all the surgical inpatients. The system screening strategy considered the temperature, positive microbiologic examinations, new antibiotic administration after surgery, and the surgeon’s report of SSI. This information system was evaluated in a prior study [4, 5]. Suspected SSI was automatically identified by the information system. Infection control professionals (ICPs) observed the inpatients with suspected SSI screened by the system at the bedside. The SSI diagnosis was made after discussion among the surgeons and ICPs [5]. SSIs were diagnosed according to the Centers for Disease Control and Prevention/National Healthcare Safety Network criteria [6]. There are three categories of SSI: superficial incisional SSI, which involves the skin or subcutaneous tissue; deep incisional SSI, which involves deep soft tissues (such as fascia and muscle) within the incision; and organ/space SSI, which involves any part of the anatomy (organ or space) other than the incision.The patients’ surgical and hospitalization information was automatically collected by computer (real-time data were collected from various systems such as the anesthesia information system and the laboratory information system). At 30 days postoperatively, a telephone follow-up was conducted by trained ICPs and entered into the system manually. The telephone interview [7] was performed to retrieve information related to the wound condition (pain or tenderness, localized swelling, redness, or elevated skin temperature), visits to surgeons or other physicians in the surgical hospital or community clinics for these problems, and eventual prescriptions of antibiotics or treatment of the wound. Patients with confirmed SSI should have signs and symptoms consistent with an infected wound or receive a diagnosis of SSI by a surgeon or physician.

### Risk factor analyses

The following were evaluated as categorical variables: patient age (≤60 years, 61–70 years, or > 70 years), body mass index (≤24 or > 24 kg/m^2^), the American Society of Anesthesiologists (ASA) score (1–2 or ≥ 3), and the duration of the operation (≤2, 2–4, or ≥ 4 h).We separated the 26 hospitals into 3 groups according to their numbers of hospital beds: 9 hospitals had ≤1500 beds, 9 had 1500–2500 beds, and 8 had ≥2500 beds.

### Statistical analysis

The data were statistically analyzed by SPSS 22.0, and the chi-square test was used for comparison of ratios. A *P* value of< 0.05 was considered statistically significant. Only factors with a *P* value of < 0.05 in the univariate analysis were entered into the multivariate analysis.

## Results

### Incidence and type of SSI

In total, 5729 patients were enrolled for the two types of surgery, and SSI occurred in 206 patients (overall incidence of 3.60%). The incidence of SSI after radical resection of rectal carcinoma was 2.01 times higher (*P* < 0.0001) than that after radical resection of colon carcinoma.

Comparison between radical resection of colon versus rectal carcinoma revealed a superficial incisional SSI rate of 0.94% versus 2.28% (*P* < 0.0001), deep incisional SSI rate of 0.56% versus 1.11% (*P* = 0.018), and organ/space SSI rate of 1.06% versus 1.72% (*P* = 0.031), respectively (Table 1).

**Table 1:** The comparison between incidence and type of SSIs in two types of surgery

SSIs type	Radical resection of colon (*n* = 3406)		Radical resection of rectal (*n* = 2323)		*X* ^*2*^	*P* value
	n	%	n	%		
Total SSIs	87	2.55	119	5.12	26.281	0.000
Superficial incisional SSI	32	0.94	53	2.28	17.017	0.000
Deep incisional SSI	19	0.56	26	1.11	5.585	0.018
Organ/space incisional SSI	36	1.06	40	1.72	4.665	0.031

### Postoperative SSI

The proportion of patients successfully contacted for telephone follow-up was 87.99%. The follow-up showed that after hospital discharge, SSI occurred in 21 (24.14%, 21/87) patients who had undergone radical resection of colon carcinoma and in 25 (21.01%, 25/119) patients who had undergone radical resection of rectal carcinoma. The average time for SSI occurrence after hospital discharge was 5 days for radical resection of colon carcinoma and 6 days for radical resection of rectal carcinoma.

### Pathogens detected in SSIs

An etiological diagnosis was conducted in 99 of 206 patients with SSIs, and 103 pathogens were detected. Thirty-eight pathogens were detected from patients who underwent radical resection of colon carcinoma, including 21 (55.26%) with *Escherichia coli*, 5 (13.16%) with *Pseudomonas aeruginosa*, and 3 (7.89%) each with *Enterococcus* spp. and *Klebsiella pneumoniae*. Sixty-five pathogens were detected from patients who underwent radical resection of rectal carcinoma, including 24 (36.92%) with *Escherichia coli*, 14 (21.54%) with *Enterococcus* spp., and 4 (6.15%) each with *P. aeruginosa* and *K. pneumoniae*. There was no significant difference in the spectrum of pathogens between the two surgical types (*P* > 0.05) (Table 2).

**Table 2:** The comparison of the common pathogens in two types of surgery

Pathogens	Radical resection of colon (*n* = 38)		Radical resection of rectal (*n* = 65)		*X* ^*2*^	*P* value
	n	%	n	%		
*Escherichia coli*	21	55.26	24	36.92	3.279	0.070
*Pseudomonas aeruginosa*	5	13.16	4	6.15	1.475	0.225
*Enterococcus*	3	7.89	14	21.54	3.239	0.072
*Klebsiella pneumoniae*	3	7.89	4	6.15	0.115	0.735

### Univariate analysis

There were no significant differences in age, sex, BMI, preoperative hospital stay, or time of antibiotic administration (0.5–2 h before the operation) between the two types of surgery. However, there was a significant difference in the type of surgery (emergency or selective) between the two groups of patients. The two types of surgery had different risk factors (Table 3).

**Table 3:** Univariate and Multivariate analysis of risk factors in patients with SSI underwent two types of surgery

Risk factor	Radical resection of colon carcinoma						Radical resection of rectal carcinoma					
	Cases	SSI(%)	Univariate analysis		Multivariate analysis		Cases	SSI(%)	Univariate analysis		Multivariate analysis	
			χ^2^	*P* Value	Odds Ratio (95%CI)	*P* Value			χ^2^	*P* Value	Odds Ratio (95%CI)	*P* Value
Age (yr)			0.935	0.627					4.101	0.129		
≤60	1555	2.38					1061	6.03				
61–70	1083	2.77					807	4.96				
> 70	723	3.04					451	3.55				
Sex			3.532	0.060					0.078	0.781		
Male	2016	2.98					1436	5.22				
Female	1390	1.94					887	4.96				
BMI			0.107	0.743					0.916	0.338		
≤24	2437	2.50					1549	4.97				
> 24	887	2.71					689	5.95				
Preoperative hospital stay			0.021	0.885					0.006	0.940		
< 48 h	222	2.70					71	4.23				
≥48 h	3184	2.54					2252	5.15				
Surgery type			5.763	0.016	1.558(0.882–2.753)	0.127			17.536	0.000	1.995(1.268–3.139)	0.003
Emergency	390	4.36					296	10.14				
Selective	3016	2.32					2027	4.39				
Operative procedure			5.446	0.020	0.540(0.344–0.847)	0.007			0.932	0.334	0.672(0.448–1.006)	0.054
Laparoscopic	1710	1.93					1628	4.85				
Open	1696	3.19					695	5.82				
ASA score			4.281	0.039	0.653(0.387–1.103)	0.111			2.700	0.100	0.588(0.361–0.958)	0.033
1–2	2907	2.34					2003	4.84				
≥3	481	3.95					312	7.05				
Operative time			25.636	0.000	0.513(0.379–0.692)	0.000			5.802	0.122	0.619(0.472–0.812)	0.001
≤2 h	724	1.80					294	3.06				
2–4 h	2156	2.04					1410	4.89				
≥4 h	526	5.70					619	6.62				
0.5-2 h medication			0.332	0.564					0.760	0.383		
Yes	2038	2.45					1108	5.60				
No	1544	2.79					1130	4.78				
No. of beds			5.816	0.055	0.644(0.451–0.921)	0.016			14.707	0.001	0.513(0.356–0.739)	0.000
≤1500	591	1.35					274	2.19				
1500–2500	1297	2.39					655	3.36				
≥2500	1518	3.16					1394	6.53				

### Logistic multivariate regression analysis

The multivariate logistic regression analysis showed that the operating time and number of hospital beds were common risk factors for SSI in the two types of surgery (Table 3). Laparoscopic surgery was an independent protective factor for radical resection of colon carcinoma. The type of emergency surgery and a high ASA score were independent risk factors for SSI after radical resection of rectal carcinoma.

## Discussion

Multicenter SSI surveillance is seldom carried out in China. The only large multicenter study reported was organized by the Organization of Chinese Hospital Association [8]. This study unified the surveillance and training methods but did not limit the scope of disease or distinguish between colon and rectal surgery; it showed that the incidence of SSI after colorectal surgery was 4.47% [8]. The present study involved 26 hospitals with different numbers of beds in 14 cities of China. Surveillance was applied to 5729 patients who underwent two types of surgery, and data were monitored and collected by an information system to ensure the accuracy of the data. The present study referred to other multicenter surveillance studies conducted worldwide. For example, Pendlimari [9] reported that SSI surveillance of colorectal surgery should take the disease classification into account because the risk factors for SSI differ significantly for ulcerative colitis, benign tumors, colon cancer, and rectal cancer. In our study, surgery with broad surveillance was avoided (such as large bowel surgery), and the procedure code and disease classification were precisely restricted by theICD-9-CM-3 and ICD-10, respectively. Each surgical procedure corresponding to a single disease, such as radical resection of colon carcinoma, was only conducted in patients with colon cancer. Additionally, the surveillance clearly defined the radical resection ranges for procedures in the ICD-9-CM-3 and excluded other nonradical operations such as exploratory laparotomy or fistulation.

The proportion of patients successfully contacted for telephone follow-up was 87.99%, which ensured surveillance of infections in patients after discharge and correct evaluation of the incidence of SSI [10, 11]. The proportion of SSIs after hospital discharge was 21.84%, which differed from the 9.52% (2/21) rate of total SSIs after colorectal resection happened during discharge [12]. We showed that the average time of occurrence of SSI after hospital discharge was 5 days for radical resection of colon cancer and 6 days for radical resection of rectal cancer. Guo [13] showed that 63.41% of patients who underwent gastrointestinal surgery in one Chinese tertiary hospital managed their wounds by themselves or received assistance from their family members, while 70.29% of patients who managed their wounds at home were not sure of whether they had received guidance and training in wound care. There is a high incidence of SSI after discharge following radical resection of colon or rectal carcinoma, emphasizing the importance of patient education for wound care in surgical hospitals.

The surgical procedure and postoperative treatment differ depending on the underlying disease; therefore, the incidence of SSI in different procedures may vary from disease to disease [9]. The present study showed that the incidence of SSI after radical resection of rectal cancer was 5.12%, which was twice as high as that after radical resection of colon cancer (2.55%). Additionally, comparison of the colon versus rectal groups revealed a superficial incisional SSI rate of 0.94% versus 2.28% (*P <* 0.0001), deep incisional SSI rate of 0.56% versus 1.11% (*P* = 0.018), and organ/space SSI rate of 1.06% versus 1.72% (*P* = 0.031), respectively. The incidence of SSI in the present study differed from that reported previously. The American College of Surgeons National Surgical Quality Improvement Program (ACS NSQIP) reported an incidence of 4.0% [14], and Public Health England reported an incidence of 8.8% in large bowel surgery in 47 hospitals [1]. These differences might be because the studies had different operating times, disease types, surgical techniques, and surgical management. Our multivariate logistic regression analysis showed that the operating time and number of hospital beds were common risk factors for SSI in the two types of surgery. The type of emergency surgery and a high ASA score were independent risk factors for SSI after radical resection of rectal carcinoma. Laparoscopic surgery was an independent protective factor after radical resection of colon carcinoma, but not for rectal surgery. The use of laparoscopy in colon and rectal surgery is associated with a minimally invasive approach, which may also be associated with a reduced risk of SSI. Most studies have shown that laparoscopic surgery is an independent protective factor for colorectal surgery [15, 16]. Other studies comparing SSI after laparoscopic surgery versus open surgery have reported controversial results [17, 18]. The incidence of SSI after laparoscopic or open rectal surgery was 4.85 and 5.82%, respectively, with no significant difference between the two groups. The small number of cases in the laparoscopic rectal surgery group might be one explanation for this lack of significance; thus, larger studies are required to clarify this issue.

One hundred three pathogens were isolated from SSIs after radical resection of colorectal cancer in the present study. In total, 21 (55.26%) *Escherichia coli* and 5 (13.16%) *P. aeruginosa* pathogens were isolated from patients who underwent radical resection of colon carcinoma, and 24 (36.92%) *Escherichia coli* and 14(21.54%) *Enterococcus* pathogens were isolated from patients who underwent radical resection of rectal carcinoma. The most common pathogen was *Escherichia coli* in both types of surgery. This result is consistent with the Chinese national SSI surveillance study reported by Zhang [8], in which *Escherichia coli* (45.33%) and *Enterococcus* spp. (12.0%) were the two most common pathogens.

Limitations of this study included the fact that it was conducted within 26 hospitals, and the possible impact of individual surgeons in different hospital as a factor affecting SSI was not available. However, the importance of culturing wound secretions and the antiseptic techniques used when sampling the wound secretions were emphasized in the training sessions. In fact, the proportion of sampling for cultures in patients with SSIs was not high in the studied hospitals, and it was difficult to distinguish colonizers and pathogens. Additionally, the sample size of the laparoscopic rectal surgery group was relatively small. When feasible, we will select more laparoscopic rectal surgeries to study.

## Conclusion

The incidence of SSIs, including all three types of infection and some of their risk factors, differed between radical resection of rectal carcinoma and colon carcinoma. We suggest that SSI surveillance should be carried out according to the disease and type of surgery to obtain more specific results that will be effective in reducing SSI.

## Abbreviations

ACS NSQIP: American College of Surgeons National Surgical Quality Improvement Program
ASA: American Society of Anesthesiologists
BMI: Body mass index
CDC/NHSN: The Centers for Disease Control and Prevention-National Healthcare Safety Network, USA
ICD-10: International Classification of Diseases, 10th revision
ICD-9-CM-3: International Classification of Diseases, 9th revision; Clinical Modification of Operations and Procedures
ICPs: Infection control professionals
SSI: Surgical site infections
